# The multigenerational effects of water contamination and endocrine disrupting chemicals on the fitness of *Drosophila melanogaster*

**DOI:** 10.1002/ece3.3172

**Published:** 2017-07-12

**Authors:** Suany Quesada‐Calderón, Leonardo Daniel Bacigalupe, Andrés Fernando Toro‐Vélez, Carlos Arturo Madera‐Parra, Miguel Ricardo Peña‐Varón, Heiber Cárdenas‐Henao

**Affiliations:** ^1^ Instituto de Ciencias Ambientales y Evolutivas Facultad de Ciencias Universidad Austral de Chile Valdivia Chile; ^2^ Doctorado en ciencias, mención Ecología y Evolución Universidad Austral de Chile Valdivia Chile; ^3^ Grupo de Saneamiento Ambiental Instituto Cinara Universidad del Valle Cali Colombia; ^4^ Escuela EIDENAR‐Facultad de Ingeniería Universidad del Valle Cali Colombia; ^5^ Sección de Genética‐Departamento de Biología Universidad del Valle Cali Colombia

**Keywords:** contamination, development, *Drosophila melanogaster*, endocrine disrupting chemicals, fertility, fitness

## Abstract

Water pollution due to human activities produces sedimentation, excessive nutrients, and toxic chemicals, and this, in turn, has an effect on the normal endocrine functioning of living beings. Overall, water pollution may affect some components of the fitness of organisms (e.g., developmental time and fertility). Some toxic compounds found in polluted waters are known as endocrine disruptors (ED), and among these are nonhalogenated phenolic chemicals such as bisphenol A and nonylphenol. To evaluate the effect of nonhalogenated phenolic chemicals on the endocrine system, we subjected two generations (F0 and F1) of *Drosophila melanogaster* to different concentrations of ED. Specifically, treatments involved wastewater, which had the highest level of ED (bisphenol A and nonylphenol) and treated wastewater from a constructed *Heliconia psittacorum* wetland with horizontal subsurface water flow (He); the treated wastewater was the treatment with the lowest level of ED. We evaluated the development time from egg to pupa and from pupa to adult as well as fertility. The results show that for individuals exposed to treated wastewater, the developmental time from egg to pupae was shorter in individuals of the F1 generation than in the F0 generation. Additionally, the time from pupae to adult was longer for flies growing in the *H. psittacorum* treated wastewater. Furthermore, fertility was lower in the F1 generation than in the F0 generation. Although different concentrations of bisphenol A and nonylphenol had no significant effect on the components of fitness of *D. melanogaster* (developmental time and fertility), there was a trend across generations, likely as a result of selection imposed on the flies. It is possible that the flies developed different strategies to avoid the effects of the various environmental stressors.

## 1 INTRODUCTION

Human‐mediated land‐use change, species introductions, pollution, and ultimately climate change have caused an almost irreversible deterioration of natural environments (Dawson, Jackson, House, Prentice, & Mace, 2011; Hunter, 2007; Pereira et al., 2010). In particular, human alteration of natural environments has generated a massive amount of water pollution, which may contain a large amount of sediments, excess nutrients, and toxic chemicals (Kareiva & Marvier, 2011). Among the many toxic and carcinogenic pollutants, endocrine disruptors (ED) are known to affect the physiological pathways associated with reproduction and development of animals and plants (Guillette, 2006; Liao, Yen, & Wang, 2009; Qiu, Wang, & Zhou, 2013; Wang et al., 2015). The molecular structure of ED allows them to behave like hormones or hormone precursors (Guillette & Gunderson, 2001; Lee & Choi, 2007). As such, these disruptors have been shown to affect not only different reproductive stages but also different developmental stages of vertebrates (Guillette & Edwards, 2005; Segner et al., 2003). For example, it has been shown that ED can cause infertility due to malformations of the reproductive tract (Guillette, 2006), impaired release of pheromones (Anway & Skinner, 2008; Colborn, Vom Saal, & Soto, 1993; Guillette & Edwards, 2005; Segner et al., 2003), premature release of progesterone and progestin and occasional premature birth (Guillette, 2006; Longnecker, Klebanoff, Zhou, & Brock, 2001), changes in the development of oocytes, thyroid abnormalities and changes during molting (Depledge & Billinghurst, 1999; Ha & Choi, 2008; Hutchinson, 2002; Planelló, Martínez‐Guitarte, & Morcillo, 2008; Rodríguez, Medesani, & Fingerman, 2007), and impaired immune functioning (Martineau et al., 1988). Experiments involving ED are complicated due to the fact that EDs often naturally come from multiple sources and can generate additive effects. In particular, we used developmental time (from egg to pupae and from pupae to adult) and fertility as components of fitness, and bisphenol A (BPA) and nonylphenol (NP) were used simultaneously as ED. We obtained different concentrations of both BPA and NP by passing wastewater through different Horizontal Subsurface Flow Constructed systems. These ED were chosen for the experiments because they have characteristics (e.g., persistent, lipophilic, low vapor pressures) which facilitate dispersion in liquid environments; as such, BPA and NP have been known to contaminate groundwater reservoirs (Colborn et al., 1993; Kareiva & Marvier, 2011; Theis & Tomkin, 2012).

Although several studies have evaluated the effects of ED on animal fitness, most were focused on understanding the consequences of one endocrine disruptor at a time, and very few have looked at the combined effects of multiple ED on a given system. This is important as multiple ED are often found together in natural environments as a result of various sources of anthropogenic water pollution (Kareiva & Marvier, 2011; Theis & Tomkin, 2012). In addition, it is unknown whether organisms can evolve as a response to ED present in their environment. Given the complexity of treating contaminated waters together with the long term persistence of ED, changes in development and reproduction time might likely be the only solution for organisms that face permanent (and potentially increased) water contamination. Therefore, in this work we evaluated the impact that different concentrations of ED have on *Drosophila* development and reproduction.

## 2 MATERIALS AND METHODS

### 2.1 Experimental design

**Figure 1:**
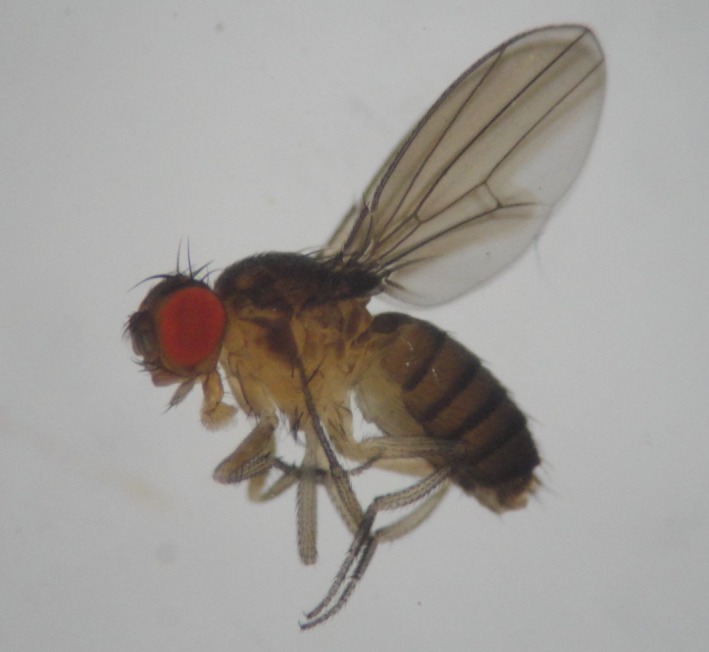
Photograph of a female specimen of *Drosophila melanogaster* (ebony mutation) from the *Drosophila melanogaster* lab at the Universidad del Valle, (Cali, Colombia)

Twenty pairs of *Drosophila melanogaster* ebony mutation (Figure 1) were used as the parental generation for the whole experiment. The flies came from lineages that have been maintained for almost 20 years at the Universidad del Valle (Sección de Genética, Departamento de Biología, Colombia).

**Figure 2:**
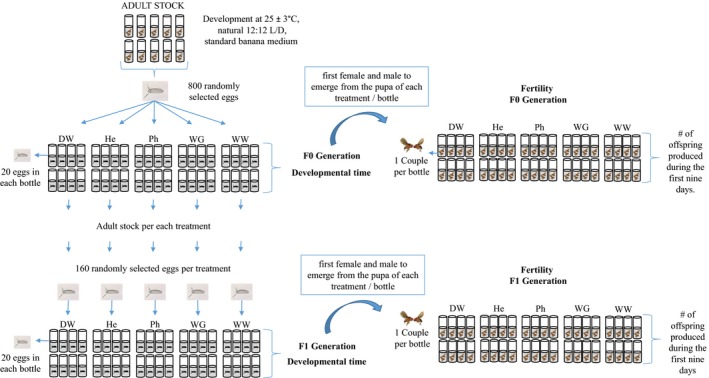
Experimental design and treatment scheme. The experimental treatments were: DW (Distilled water), He (Treated wastewater from a HSSF‐CW with *Heliconia psittacorum*), Ph (Treated wastewater from a HSSF‐CW with *Phragmites australis*), WG (Treated wastewater from a gravel HSSF‐CW), WW (Wastewater)

The flies were kept in a temperature controlled room at 25 ± 3°C with a natural 12L:12D photoperiod and were fed on a standard banana medium prepared with 150 ml of sterilized water from each of the following five experimental treatments (Figure 2). (1) Wastewater from a natural environment, this wastewater came from the aqueduct of the city of Ginebra (Valle del Cauca, Colombia) and had a concentration of 8.8 ± 6.4 μg/L (mean ± 1 *SD*) of bisphenol A (BPA) and a concentration of 1670.9 μg/L ± 837.6 g/L (mean ± 1 *SD*) of nonylphenol (NP). Endocrine disrupting chemicals (EDCs) were determined using stir bar sorptive extraction in line with thermal desorption and gas chromatography/mass spectrometry (TDU‐GC–MS) at University of Texas at El Paso. (2) Treated wastewater from a constructed *Heliconia psittacorum* wetland with horizontal subsurface water flow (HSSF‐CW; Ascuntar Ríos, Toro Vélez, Peña, & Madera Parra, 2009; Toro‐Vélez et al., 2016). *Heliconia psittacorum* is a native plant of the Caribbean and South America that is known to have a high phytoremediation capacity and is commonly used to treat domestic wastewater in Colombia (Peña‐Salamanca, Madera‐Parra, Sánchez, & Medina‐Vásquez, 2013). Likewise, this treated wastewater had a BPA concentration of 1.4 ± 0.71 μg/L (mean ± *SD*) and a NP concentration of 629.3  ± 318.4 μg/L (mean ± *SD*) which is 79.3% less BPA and 62.8% less NP than that of wastewater. (3) Treated wastewater from a HSSF‐CW with *Phragmites australis*,* P. australis* is a cosmopolitan perennial plant commonly used in the treatment of domestic wastewater (Zhi & Ji, 2012). Water from this treatment had a BPA concentration of 1.58 ± 0.85 μg/L (mean ± *SD*) and a NP concentration of 735.9  ± 283.6 μg/L (mean ± *SD*) which is 76% less BPA and 25.3% less NP than that of wastewater. (4) Treated wastewater from a gravel HSSF‐CW that had a BPA concentration 2.64 ± 0.65 μg/L (mean ± *SD*) and a NP concentration of 1252.5 ± 540.62 μg/L (mean ± *SD*) which is 70% less BPA and 25% less NP than that of WW. (5) Distilled water (without EDCs and non‐polluted sources).

### 2.2 Developmental time

The 20 pairs of flies were left to reproduce in 10 bottles containing standard banana medium. After 4 days, adults were discarded and all the eggs from all the flies were collected and mixed. We randomly selected 800 eggs that were transferred to 40 bottles (Figure 2), which is eight bottles per experimental treatment, each with 20 eggs. All bottles were kept at 25 ± 3°C with a natural 12L:12D photoperiod.

Developmental time was estimated as the time in days it takes an egg to become pupae and the time it takes a pupae to become adult. Every day the number of pupae in each bottle was counted and, to prevent counting the same individual twice, a mark on the bottle was made. The number of emerging adults was counted on a daily basis until no more adults emerged. The pair that emerged first from each bottle and treatment was used for the fertility experiment. The remaining flies from each experimental treatment were allowed to reproduce in 10 bottles with the corresponding experimental medium (Figure 2). After 4 days, all eggs (i.e., F1 generation) from each treatment (160 each treatment) were collected and mixed in order to maintain randomness in each treatment. These were then transferred to eight new bottles, and developmental times were estimated as before.

### 2.3 Fertility

The first emerging adult couple from each bottle from each treatment was allowed to reproduce in a new bottle with the corresponding experimental medium. After 9 days, the couple was discarded and the number of adults was counted until no more adults emerged. This was repeated for the F1 generation.

### 2.4 Statistical analyses

Two‐way ANOVAs were used to evaluate how experimental treatment, generation, and their interaction affected developmental time (egg to pupae and pupae to adult). Data per bottle were averaged before analyses and response variables were log10‐transformed to meet parametric assumptions of homoscedasticity and normality. Each experimental treatment had eight independent replicates. Nonsignificant terms were removed from the final model. Tukey's HSD was employed as a posteriori test. A generalized linear model with a quasi‐Poisson error was used to evaluate how experimental treatment, generation, and their interaction affected fertility. All analyses were carried out in R v3.1.3 (Team R Core, 2015).

## 3 RESULTS

### 3.1 Developmental time

**Figure 3:**
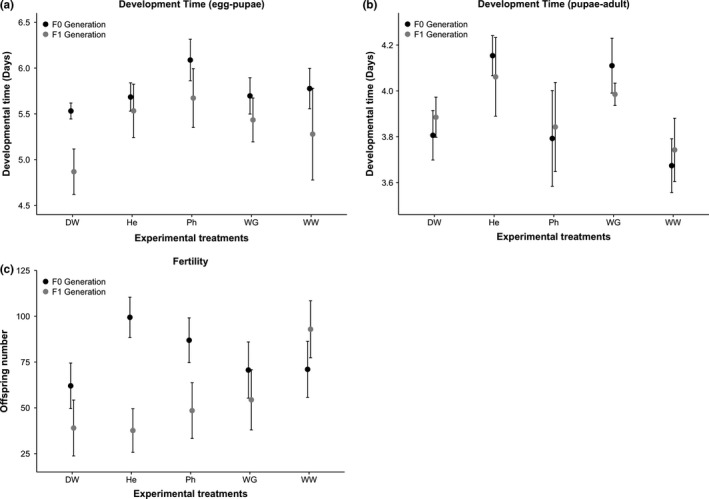
Response of *Drosophila melanogaster* exposed to five experimental treatments over two generations (F0 generation with black dots and F1 with gray dots). (a) Average (± *SE*) developmental time from egg to pupae for both generations. (b) Average (± *SE*) developmental time from pupae to adult for both generations. (c) Fertility is represented by the average (± *SE*) number of offspring produced during the first 9 days in each generation. The experimental treatments are shown on the *x*‐axis, DW: Distilled water, He: Treated wastewater from a HSSF‐CW with *Heliconia psittacorum*, Ph: Treated wastewater from a HSSF‐CW with *Phragmites australis*, WG: Treated wastewater from a gravel HSSF‐CW, WW: Wastewater

Developmental time from egg to pupae was not affected by the interaction between experimental treatment and generation (*F*_4,70_ = 0.40, *p *=* *.808) nor was it affected by the experimental treatment itself (*F*_4,74_ = 1.79, *p *=* *.140). However, developmental time from egg to pupae was shorter for individuals of the F1 generation (*F*_1,74_ = 7.73, *p *=* *.007) (Table 1; Figure 3a).

**Table 1:** Summary of the statistical analyses evaluating differences in *Drosophila melanogaster* development and reproduction. Individuals were exposed to one of five experimental treatments over two generations (F0 and F1). Significant values are in bold (*p* < .05)

	S.S	*df*	M.S	*F*	*p*‐Value
Developmental time (Egg—Pupae)					
Treatment	0.0231	4	0.0058	1.7300	.1532
Generation	0.0250	1	0.0250	7.4780	**.0079**
Interaction	0.0054	4	0.0013	0.4010	.8075
Residuals	0.2341	70	0.0033	—	—
Developmental time (Pupae—Adult)					
Treatment	0.0236	4	0.0059	2.8540	**.0299**
Generation	0.0000	1	0.0000	0.0002	.9898
Interaction	0.0019	4	0.0005	0.2280	.9220
Residuals	0.1445	70	0.0021	—	—
Fertility					
Treatment	8,246	4	2,061	1.2820	.2853
Generation	11,045	1	11,045	6.8710	**.0107**
Interaction	15,184	4	3,796	2.3610	.0601
Residuals	112,531	70	1,608	—	—

Although developmental time from pupae to adults was not affected by the interaction between experimental treatment and generation (*F*_4,70_ = 0.23, *p *=* *.922) nor was it affected by generation alone (*F*_1,74_ = 0.001, *p *=* *.990), it was affected by the experimental treatment (*F*_4,74_ = 2.98, *p *=* *.025). In particular, developmental time was longer for flies growing in the *H. psittacorum‐*treated water (Figure 3b).

### 3.2 Fertility

Fertility was not affected by the interaction between experimental treatment and generation ($\chi_{\left[4\right]}^{2}$ = 234.1, *p *=* *.072). Fertility was also not affected by experimental treatment ($\chi_{\left[1\right]}^{2}$ = 125.7, *p *=* *.341). However, fertility was higher in the F0 generation ($\chi_{\left[1\right]}^{2}$ = 167.7, *p *=* *.014) (Figure 3c).

## 4 DISCUSSION

Water pollution as a result of anthropogenic activities (e.g., sediment, excess nutrients, and toxic chemicals) can negatively alter freshwater ecosystems. Endocrine disruptors (ED) are known to interfere in different ways with the normal synthesis, secretion, transport, and action of hormones (Guillette, 2006; Segner et al., 2003). Furthermore, some studies have indicated that the biodiversity of freshwater ecosystems is particularly vulnerable to ED (Cardinale et al., 2012; Kareiva & Marvier, 2011). Here, we evaluated the impact that different concentrations of ED have on components of *Drosophila melanogaster*'s fitness (developmental time and fertility) over two generations. Our results show that: (1) the developmental time from egg to pupae was shorter for individuals of the F1 generation, (2) developmental time from pupae to adult was longer for flies growing in *H. psittacorum* treated wastewater, and (3) fertility was lower in the F1 generation.

A major selective force in nature is exposure to environmental perturbations (Bijlsma & Loeschcke, 2005; Hoffmann & Hercus, 2000; Hoffmann & Parsons, 1991). Animals have evolved several strategies, from genetic (*e.g.,* Alvarez, Espinoza, Inostroza B, & Arce, 2015; Hebbelmann et al., 2012; Lardies, Arias, & Bacigalupe, 2010; Silva, Bacigalupe, Luna‐Rudloff, & Figueroa, 2012; Sørensen & Loeschcke, 2004), to physiological (*e.g.,* Bozinovic, Catalán, & Kalergis, 2013; Castañeda et al., 2011; Chapin, Autumn, & Pugnaire, 2012; Hermes‐Lima & Zenteno‐Savìn, 2002; Uy, Leduc, Ganote, & Price, 2015) and behavioral (*e.g.,* Kitaysky, Wingfield, & Piatt, 2001; Koolhaas et al., 1999; Ruiz‐Aravena et al., 2014; Wingfield & Kitaysky, 2002) to deal with or avoid the effects of such stressors. Additionally, fitness‐related traits are not an exception and are also affected by environmental stress (Bijlsma & Loeschcke, 2005). Nevertheless, there is no clear pattern regarding whether fitness should increase or decrease in the face of environmental stress. Our experimental treatments with different concentrations of BPA and NP did not produce any effect on the *D. melanogaster* fitness components. However, the means of the measured fitness components (developmental time and fertility) were significantly different between generation F0 and F1; this observed response was likely a result of the selection imposed on the flies.

Although several studies have shown that developmental time in *Drosophila* species is affected by environmental stress (Castañeda & Nespolo, 2013; Sørensen & Loeschcke, 2004), there is no clear pattern regarding the effect of ED. While some studies have shown that developmental time increases from pupae to adult when flies are exposed to ED (Akins, Schroeder, Brower, & Aposhian, 1992; Cohn, Widzowski, & Cory‐Slechta, 1992; Liu, Li, Prasifka, Jurenka, & Bonning, 2008), other studies report that developmental time from pupae to adult decreases (Atli, 2010; Memmi & Atlı, 2009). Our results do not provide any new insight regarding the consequences of exposure to ED on components of fitness measured here: Developmental time was not significantly influenced by wastewater. Furthermore, there was no consistent pattern (i.e., in treatment and/or generation) between developmental time from egg to pupae and developmental time from pupae to adult (Figure 3). The reasons for that unconsistency remain unclear. As the second emerging couples (i.e., second shortest developmental time) were the parents of the F1 generation, developmental time from egg to pupae was only shorter in the F1 generation. In contrast to the results found here, some studies have shown that exposure to ED in invertebrates causes overall reduced growth rates and thus, overall increases in developmental time (Hill et al., 2002; Izumi, Yanagibori, Shigeno, & Sajiki, 2007; Marcial, Hagiwara, & Snell, 2003), and yet other studies have shown the opposite pattern increased growth rates and decreased development time (Weiner et al., 2014).

Wastewater contains other estrogenic EDs such as ethinelestradiol (EE2) and phthalate esters (e.g., dibutyl phthalate). The effect of these EDs could also affect the generation time of *D. melanogaster* (Memmi & Atlı, 2009) or of other invertebrates such as *Brachionus calyciflorus* and *Haliotis diversicolor* (Huang, Sun, & Song, 1999; Zhao, Xi, Huang, & Zha, 2009) Zhao et al., 2009). Thus, despite *H. pisttacorum*′s ability to remove ED (Ascuntar Ríos et al., 2009; Peña‐Salamanca et al., 2013), the treated wastewater could still contain these or other EDs in low amounts; this in turn would explain why the HSSF‐CW *H. psittacorum*‐treated wastewater, with lower levels of BPA and NP, had an effect on development time.

Unfortunately, the patterns are not clear regarding fertility: Some studies have shown that exposure to ED reduces fertility (Atli, 2013; Atli & Unlu, 2012; Liu, Li, Zhao, Zhang, & Gu, 2014; Mihaich et al., 2009), increases fertility (Marcial et al., 2003; Widarto, Krogh, & Forbes, 2007) or has not produced any (significant) effect (Forbes, Warbritton, Aufderheide, Van Der Hoeven, & Caspers, 2008; Forget‐Leray, Landriau, Minier, & Leboulenger, 2005). Again, our results do not offer a strong signal on the consequences of ED exposure, as fertility was only affected by generation (i.e., lower in F1 generation) but not by experimental treatment. However, our results are in agreement with a trade‐off between faster developmental rates and lower fertility (Nunney, 1996; Roff, 2001). In *Drosophila*, selection for faster developmental rates, which is what we have indirectly carried out in order to obtain the F1 generation, usually results in the evolution of smaller body size as adults (Chippendale & Sorenson, 1997; Chippindale, Chu, & Rose, 1996). As body size is positively related to fertility (Roff, 2001), individuals that develop faster are smaller and thus have less offspring.

In conclusion, it is well‐known that anthropogenic effects on ecosystems, such as contamination of water with toxic chemicals, such as EDs, have an effect on the fitness of individuals (Anway & Skinner, 2008; Colborn et al., 1993; Depledge & Billinghurst, 1999; Weiner et al., 2014). Despite this, the present study did not find a significant effect of EDs (i.e., BPA and NP) on the developmental time or fertility of two generations of *D. melanogaster*. Overall, more studies are needed to evaluate the generality of this finding. Studies of morphological variation and studies involving many generations will help to determine how *D. melanogaster* is affected by EDs.

## CONFLICT OF INTEREST

None declared.
